# Are intestinal helminths playing a positive role in tuberculosis risk? A systematic review and meta-analysis

**DOI:** 10.1371/journal.pone.0223722

**Published:** 2019-10-15

**Authors:** Ali Taghipour, Mehrdad Mosadegh, Fatemeh Kheirollahzadeh, Meysam Olfatifar, Hossein Safari, Mohammad Javad Nasiri, Atefeh Fathi, Milad Badri, Hadi Piri Dogaheh, Taher Azimi

**Affiliations:** 1 Department of Parasitology, Faculty of Medical Sciences, Tarbiat Modares University, Tehran, Iran; 2 Department of Pathobiology, School of Public Health, Tehran University of Medical Sciences, Tehran, Iran; 3 Biology Department, School of Basic Science, Science and Research Branch Islamic Azad University (SRBIAU), Poonak, Tehran, Iran; 4 Gastroenterology and Liver Diseases Research Center, Research Institute for Gastroenterology and Liver Diseases, Shahid Beheshti University of Medical Sciences, Tehran, Iran; 5 Health Promotion Research Center, Iran University of Medical Sciences, Tehran, Iran; 6 Department of Microbiology, School of Medicine, Shahid Beheshti University of Medical Sciences, Tehran, Iran; 7 Faculty of veterinary medicine, University of Zabol, Zabol, Iran; 8 Department of Microbiology, School of Medicine, Ardabil University of Medical Science, Ardabil, Iran; University of Mississippi Medical Center, UNITED STATES

## Abstract

**Background:**

Co-infection of intestinal helminthic infections (IHIs) and tuberculosis (TB) has appeared as a public health issue, especially in developing countries. Some recent studies have been carried out on the possible relevance of IHIs to TB. The current systematic review and meta-analysis was conducted to assess the prevalence and odds ratio (OR) of IHIs among TB patients and clarify the relationship between IHIs and TB disease.

**Methods:**

For the purpose of the study, five English databases including PubMed, Science Direct, Scopus, Web of Science (ISI), and Google scholar were searched (up to January 30, 2019) in order to find the related studies. Random-effects meta-analysis model was used to estimate the pooled prevalence, odds ratio (OR), and 95% confidence interval (CI). Inclusion and exclusion criteria were applied.

**Results:**

A total of 20 studies including 10 studies with case-control design (2217 patients and 2520 controls) and 10 studies with cross-sectional design (a total of 2415 participants) met the eligibility criteria. As shown by the random-effects model, the pooled prevalence of IHIs in TB patients was estimated to be 26% (95% CI, 17–35%; 1249/4632). The risk of IHI was higher in TB patients compared to controls but this was not statistically significant. However, according to genus/species, the pooled OR of *Strongyloides stercoralis* (*S*. *stercoralis*) (OR, 2.68; 95% CI, 1.59–4.54) had a significantly higher risk in TB patients compared to controls. Nevertheless, the results of random effects model showed no statistically significant association between overall pooled OR of IHIs in TB patients compared to controls in case-control studies (OR, 1; 95% CI, 0–1).

**Conclusions:**

It is highly recommended that more precise studies should be carried out by researchers in order to better understand this association. Also, it is of great importance to include the periodic screenings for IHIs in the routine clinical care of these patients.

## 1. Introduction

Tuberculosis (TB) is the most important public health issue in regions with low level of hygiene worldwide [1, 2]. According to the latest reports by World Health Organization (WHO), there is an estimated 10 million new cases of TB and it has caused 1.3 million deaths [3]. Failure or suppression of the host’s immune system leads to the progression of Mycobacterium tuberculosis (MTB) infection and ultimately results in the development of an active form of the disease[4]. Intestinal helminthic infections (IHIs) affect the nutritional and immunological status of the host and lead to alterations in the immune response, favoring the occurrence of other bacterial infections [5–7]. Furthermore, based on the results of the several published studies, 819, 467.6, and 438.9 million individuals were estimated to be infected by *Ascaris lumbricoides*(*A*. *lumbricoides*), *Trichuris trichiura* (*T*. *trichiura*), and hookworms, respectively [8, 9]. During the last two decades, it looks as if the co-existence of IHIs and TB has appeared as a public health problem, especially in developing countries [9, 10]. IHIs is associated with the production of interleukin 4 (IL-4), IL -5, IL-9, IL-10, and IL-13 in the host and is likely to play a vital role in decreasing the severity of acute diseases caused by helminth infection [11]. On the other hand, change from T helper 1 (Th1) towards Th2 during IHIs can decrease Th1 immune response to MTB, which is often involved in the protection against intracellular pathogens. Overall, the interaction between IHIs and TB is vague and controversial, and difficult to be fully understood [12, 13]. Several studies have been carried out on IHIs among TB patients; however, there has been no complete investigation aiming to collect and systematically analyze this domain. Therefore, the present systematic review and meta-analysis aimed to assess the prevalence and odds ratio (OR) of IHIs among TB patients and clarify the relationship between IHIs and TB disease.

## 2. Methods

### 2.1. Search strategy

This systematic review and meta-analysis was prepared and reported based on the Preferred Reporting Items for Systematic Review and Meta-Analyses (PRISMA) guidelines[14]. To assess the prevalence and odds ratio (OR) of IHIs in the TB patients, a wide-ranging literature search was carried out in five English databases, including PubMed, Science Direct, Scopus, Web of Science, and Google Scholar (from their inception until January 30, 2019). We performed the searching process using the following keywords based on the medical subject heading (MeSH) terms, including: “parasite,” “intestinal parasites,” “helminth,” “tuberculosis,” “*Mycobacterium tuberculosis*,” “pulmonary tuberculosis,” “epidemiology,” and “prevalence,” alone or in combination with “OR” and/or “AND” in English language. The search terms used in five English databases are shown in S1 File.

### 2.2. Inclusion criteria

The following inclusion criteria were used in the present systematic review: (1) peer-reviewed original research papers and short reports; (2) case-control and cross-sectional studies that estimated the prevalence of IHIs in adult and children TB cohorts; (3) studies published with full text or abstracts in English; (4) studies published online in English databases from their inception until January 30, 2019; (5) studies reporting the precise total sample size and positive samples in case-control and cross-sectional studies; and (6) studies that surveyed at least one type of intestinal helminths by standard parasitological methods.

### 2.3. Exclusion criteria

The exclusion criteria included: (1) review articles, systematic reviews, editorials, letters and case reports; (2) those articles that were not available in English language and were irrelevant to the topic of interest.

### 2.4. Study selection and data extraction

The initial records gathered during database searching were saved in a Word file based on their topics and/or abstracts. After a primary screening, the potentially eligible records were selected in order to download their full-text.

Two trained researchers (A. T and M. B) assessed the final eligibility and inclusion criteria for the downloaded full texts. Then, the selected papers were read very carefully and with details. Later on, disagreements between the reviewers were resolved through discussion and consensus with a third reviewer (T. A). Furthermore, the agreement between the two reviewers was analyzed using the Kappa test. Afterwards, the necessary data were extracted by an author (A. F), and the others (M. B and T. A) rechecked them. Additionally, we hand-checked the references of the eligible papers very carefully aiming to find related articles that were not retrieved in the database searching. Eventually, we extracted and recorded the following features of each pertinent article using Excel software (Microsoft, Redmond, WA, USA): first author, country, year of publication, sample size, diagnostic method, study design, age range or mean age, number and type of helminths of infected individuals in cross-sectional studies, number and type of helminths of infected people in cases and healthy people in case-control studies.

### 2.5. Data synthesis and statistical analysis

In present study, all statistical analyses were performed using Meta for packages of R software version 3.5.1 [15]. The prevalence of IHIs in TB patients was evaluated through generation of the pooled odds ratio (OR) and 95% confidence interval (CI) using the random effects model. OR and 95% CI were calculated for each study using a two-by-two table in case-control studies. Heterogeneity between studies was assessed using I^2^ method. I^2^ values of 25%, 50% and 75% were considered as low, moderate, and high heterogeneity, respectively. On the other hand, we used the Sidik-Jonkman method to estimate the study variance (tau^2) and Hartung-Knapp method to adjust test statistics, their confidence interval and subsequently the tests degrees of freedom[16]. Moreover, small study effects and their publication bias were discerned by a funnel plot on the cornerstone of Egger’s regression test. We also used the funnel plot to check the probability of publication bias during the analysis. P-value<0.05 was considered statistically significant. Results are shown as forest plots and difference in prevalence rate of IHIs in TB patients and controls is presented by an OR and 95% CI.

## 3. Results

### 3.1. Study characteristics

**Fig 1:**
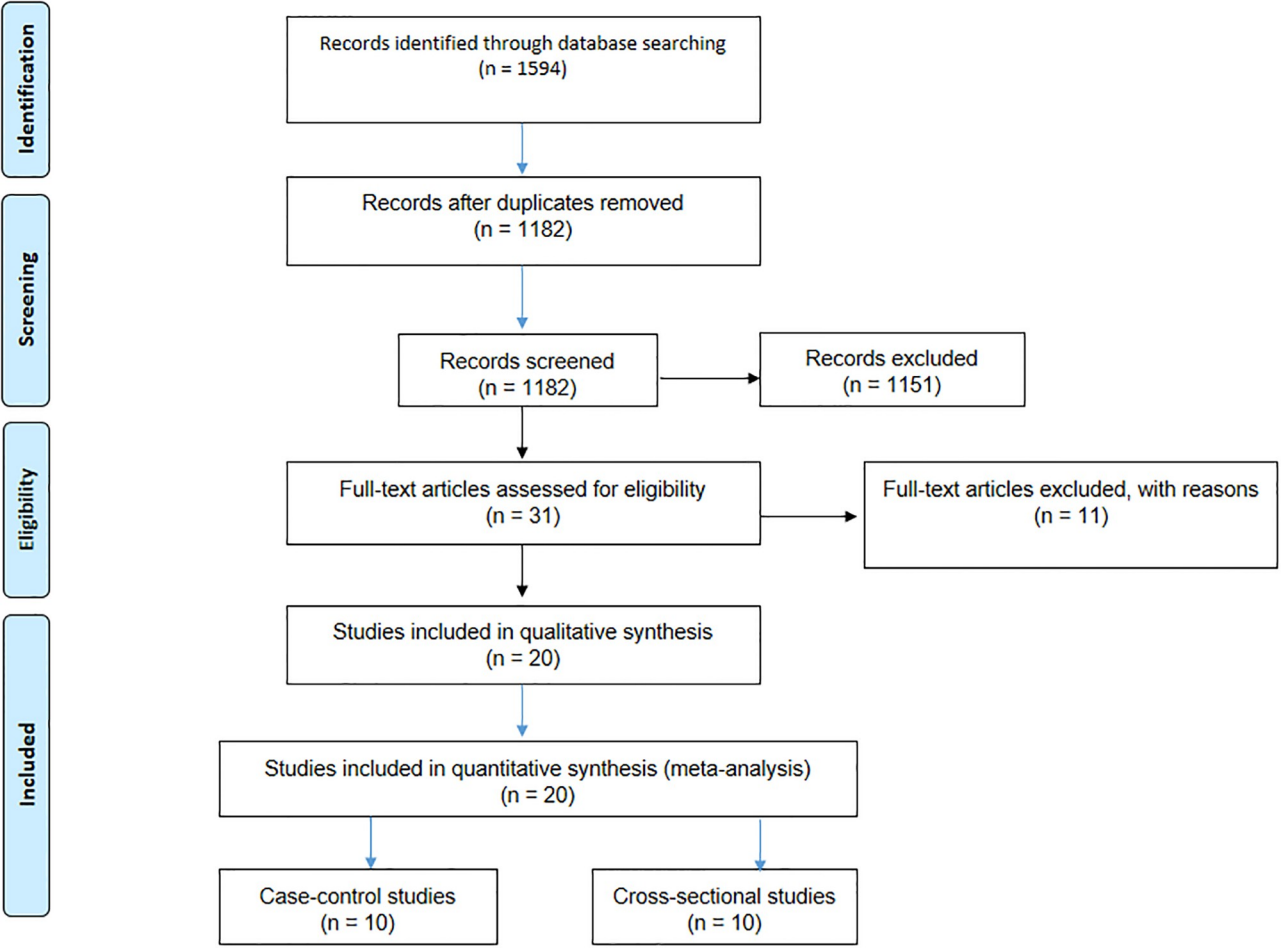
Flow diagram of the study design process.

As shown in Fig 1, we found a total of 1594 papers following the initial search of databases, and after excluding duplicates and/or non-eligible papers, 20 articles had eligibility to be considered in the current systematic review and meta-analysis [9, 12, 17–34]. The Kappa test between the reviewers showed interobserver agreement of 93.75%, which was considered to be an excellent result. Of 20 articles, 10 had a case-control design and 10 were cross-sectional. Tables 1 and 2 present the main characteristics of included studies with case-control and cross-sectional design, respectively. In case-control studies, the sample size of the cases and controls was 2217 and 2520, respectively. Case-control studies were carried out in seven diverse countries (four in Ethiopia, one in Iran, one in Brazil, one in Nigeria, one in Tanzania, one in China, and one in Peru). Cross-sectional studies, with a total of 2415 participants, were conducted in five different countries (six in Ethiopia, one in Brazil, one in Egypt, one in Iran, and one in Tanzania).

**Table 1:** Summary of studies with case-control design investigating the prevalence of IHIs in TB patients and control subjects.

First Author	Published time	Country	Methods	Age (range or mean±SD)	TB patients		Control subjects	
					N(Samples)	N(Positive)	N(Samples)	N (Positive)
**Tristao-Sa et al**.	2002	Brazil	Macroscopic and microscopic methods, Lutz-Hoffman method	NR	57	33	86	18
**Elias et al**.	2006	Ethiopia	Direct microscopy and the formal-ether concentration	10—≥50	230	163	510	185
**Marcellinus et al**.	2010	Nigeria	Macroscopic and microscopic methods and Stoll's egg counting technique	NR	96	27	156	34
**Abate et al**.	2012	Ethiopia	Direct microscopy and Kato-Katz technique	28	112	32	183	38
**Li X et al**.	2014	China	Direct microscopy and modified Kato-Katz thick smear	NR	369	28	366	30
**Franke et al**.	2014	Peru	Direct microscopy, scotch tape	6.7	189	28	189	23
**Abate et al**.	2015	Ethiopia	Direct microscopy and Kato-Katz technique	<28—≥28	306	121	306	85
**Hailu et al**.	2015	Ethiopia	Direct microscopy and the formal-ether concentration	37	100	49	168	39
**Mhimbira et al**.	2017	Tanzania	Baermann, FLOTAC, Kato-Katz, circulating cathodic antigen, urine filtration	18—≥45	597	190	375	97
**Taghipour et al**.	2019	Iran	Direct microscopy, the formal-ether concentration	≤30—≥51	161	3	181	1

**Table 2:** Summary of studies with cross-sectional design investigating the prevalence of IHIs in TB patients.

First Author	Published time	Country	Methods	Age (range or mean±SD)	TB patients	
					N (Samples)	N (Positive)
**Kassu et al**.	2004	Ethiopia	Direct microscopy and the formol-ether concentration	<40—≥40	241	110
**Ramos et al**.	2006	Ethiopia	Direct microscopy	above 12 y	100	16
**Kassu et al**.	2007	Ethiopia	Direct microscopy and the formol-ether concentration	15–50+	257	114
**Neto et al**.	2009	Brazil	Direct microscopy and Kato-Katz technique, Ritchie and Baermann	42.44 ± 12.60	327	33
**Alemayehu et al**.	2014	Ethiopia	Direct microscopy and the formol-ether concentration	<14—>45	72	21
**Alsayed Hasanain et al**.	2015	Egypt	Direct microscopy	42.7 ± 13.9	231	38
**Alemu et al**.	2017	Ethiopia	Direct saline and formol-ether concentration	15–65	213	52
**Sikalengo et al**.	2018	Tanzania	Direct microscopy, Kato-Katz and Baermann, circulating cathodic antigen, urine filtration	35	668	154
**Tegegne et al**.	2018	Ethiopia	Direct microscopy and the formol-ether concentration	5—>65	256	36
**Taghipour et al**.	2018	Iran	Direct microscopy, the formol-ether concentration	47.88 ± 10.88	50	1

### 3.2. The overall prevalence of IHIs in TB patients

**Fig 2:**
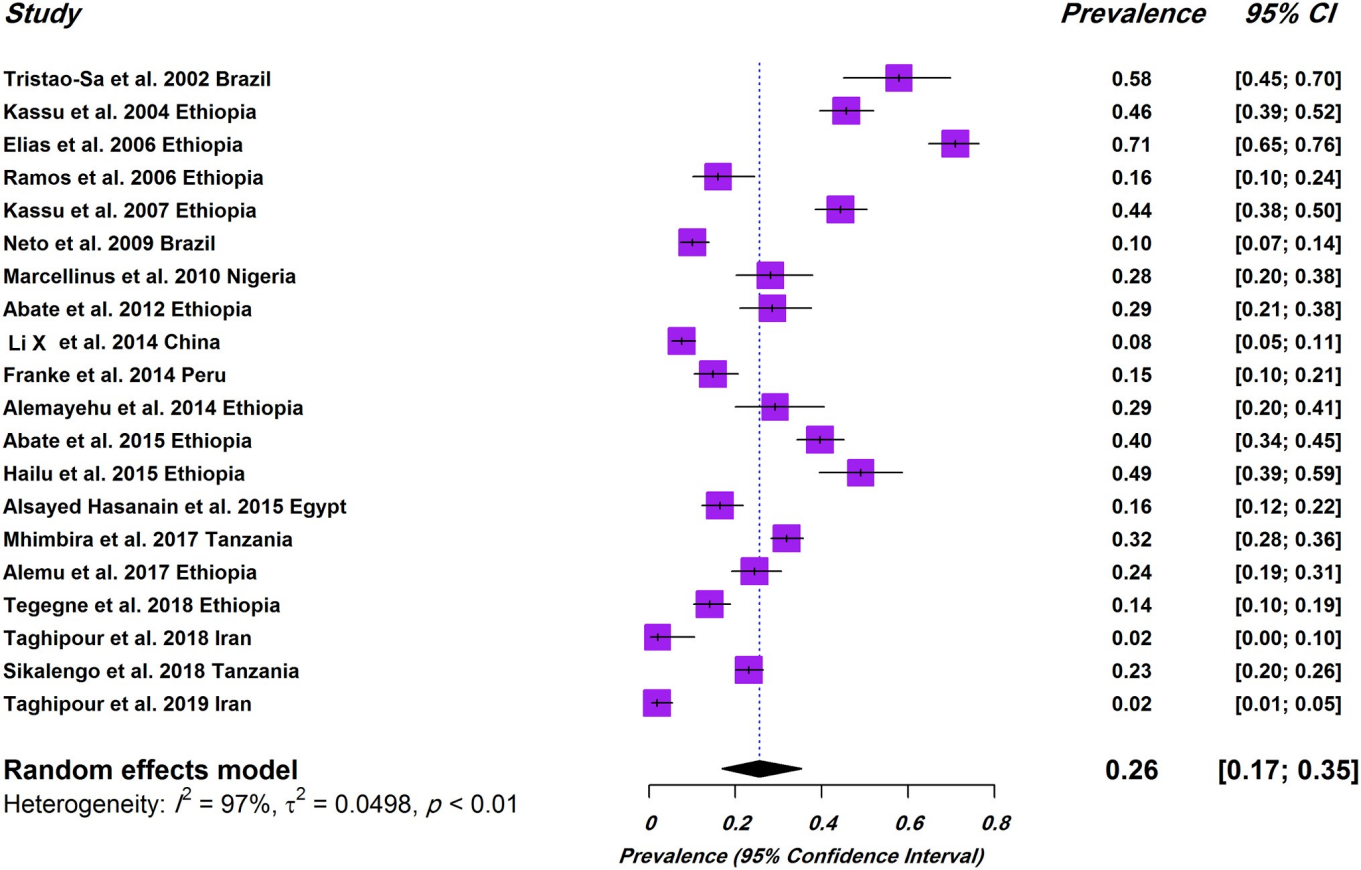
Forest plot of prevalence of intestinal helminthic infections in TB patients.

As shown by the random-effects model, the pooled prevalence of IHIs in TB patients was estimated to be 26% (95% CI, 17–35%; 1249/4632). The heterogeneity was substantial (*I*^2^ = 97%; τ^2^ = 0.049; P<0.01). The forest plot diagram of our review is illustrated in Fig 2.

#### 3.2.1. The overall prevalence/odds ratio of IHIs based on the study type

**Fig 3:**
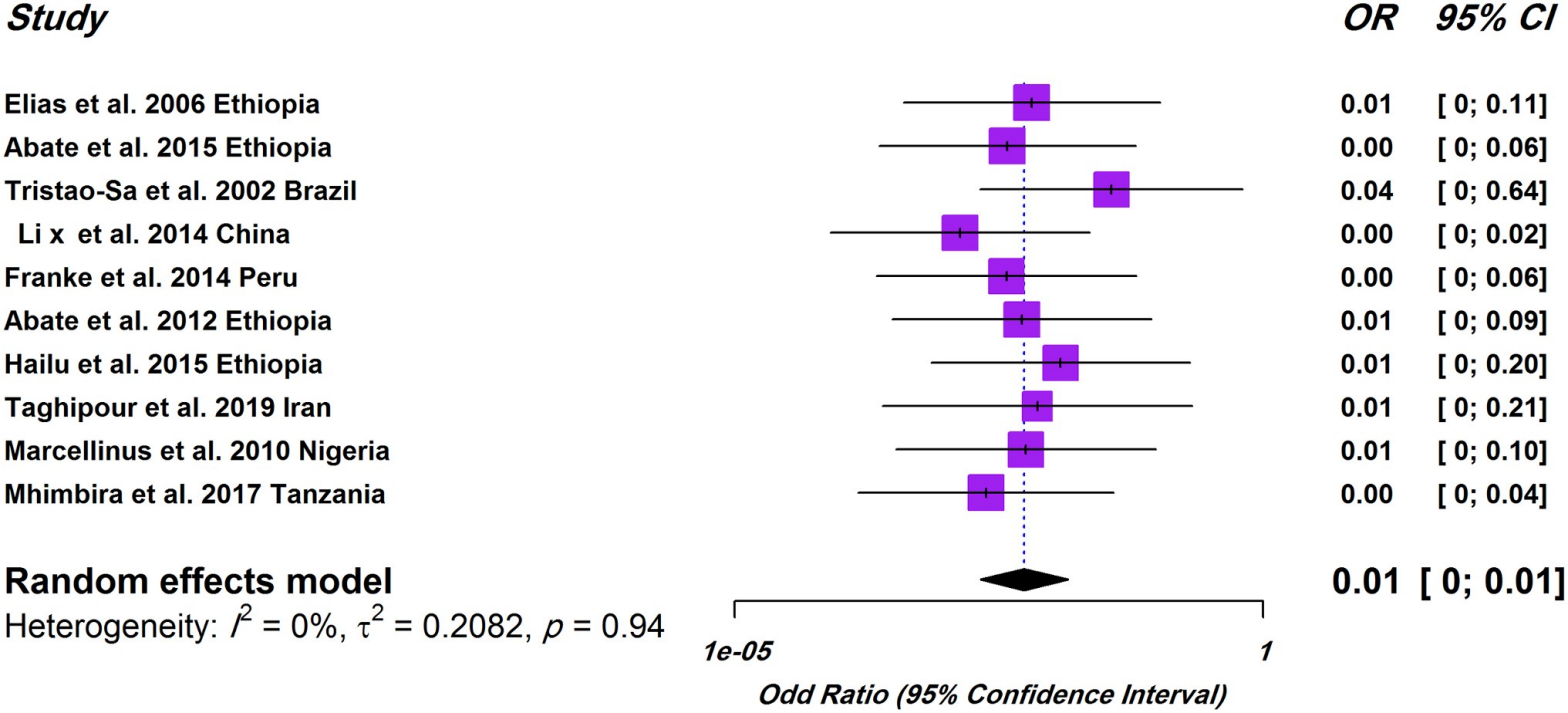
Forest plot of odds ratios for relationship between prevalence of IHIs and TB patients in case-control studies.

In case-control studies, the pooled prevalence of IHIs in TB patients was estimated to be 30% (674/2217) and its heterogeneity (*I*^2^ = 98; τ^2^ = 0.068; P<0.01) was observed (S1 Fig). Also, in cross-sectional studies, the pooled prevalence of IHIs in these patients was obtained 21% (575/2415) and its heterogeneity (*I*^2^ = 96; τ^2^ = 0.030; P<0.01) was observed (S1 Fig). As shown in Fig 3, we found that the overall pooled OR of IHIs was not significant in TB patients compared to healthy controls (OR, 1; 95% CI, 0–1) and its heterogeneity (*I*^2^ = 0; τ^2^ = 0.20; P = 0.94) was observed.

#### 3.2.2. The overall prevalence of IHIs based on the type of helminths

With regard to type of helminths in 20 studies, the estimated pooled prevalence of hookworm, *A*. *lumbricoides*, *S*. *stercoralis*, and *T*. *trichiura* was 6% (95% CI, 2–10%) with heterogeneity (*I*^2^ = 94; τ^2^ = 0.024; ρ<0.01), 7% (95% CI, 2–13%) with heterogeneity (*I*^2^ = 98; τ^2^ = 0.04; P<0.01), 5% (95% CI, 2–9%) with heterogeneity (*I*^2^ = 96; τ^2^ = 0.026; P<0.01), and 2% (95% CI, 0–3%) with heterogeneity (*I*^2^ = 83; τ^2^ = 0.008; P<0.01) among TB patients, respectively (S2–S5 Figs).

Moreover, subgroup analysis based on the type of helminths revealed the pooled OR of the higher risk of IHIs in TB patients in case-control studies, these helminths include hookworm (OR, 1.40; 95% CI, 0.93–2.12;) with heterogeneity (*I*^2^ = 56; τ^2^ = 0.09; P = 0.03), *A*. *lumbricoides* (OR, 1.48; 95% CI, 0.71–3.10) with heterogeneity (*I*^2^ = 76%; τ^2^ = 0.48; Ρ<0.01), and *T*. *trichiura* (OR, 1.96; 95% CI, 0.68–5.51) with heterogeneity (I^2^ = 64%; τ^2^ = 1.16; Ρ = 0.01) in TB patients compared to healthy controls but this was not statistically significant. However, according to genus/species, the pooled OR of *S*. *stercoralis* (OR, 2.68; 95% CI, 1.59–4.54) with heterogeneity (I^2^ = 47%; τ^2^ = 0.16; Ρ = 0.07), had a significantly higher risk in TB patients compared to controls (Table 3 and S6 and S7 Figs).

**Table 3:** Subgroup meta-analysis of the pooled OR of IHIs in case-control studies.

Helminths	No. of studies	OR (95%CI)	Heterogeneity for each subgroup		
			I^2^%	τ^2^	P
***Strongyloides stercoralis***	8	2.68 (1.59–4.54)	47	0.16	0.07
***Ascaris lumbricoides***	7	1.48 (0.71–3.10)	76	0.48	<0.01
**Hookworm**	7	1.40 (0.93–2.12)	56	0.09	0.03
***Trichuris trichuria***	7	1.96 (0.68–5.51)	64	1.16	0.01
**Total of helminths**	10	1 (0–1)	0	0	0.94

Total of helminths; all types of helminths regardless of their genus/species

### 3.3. Publication bias

**Fig 4:**
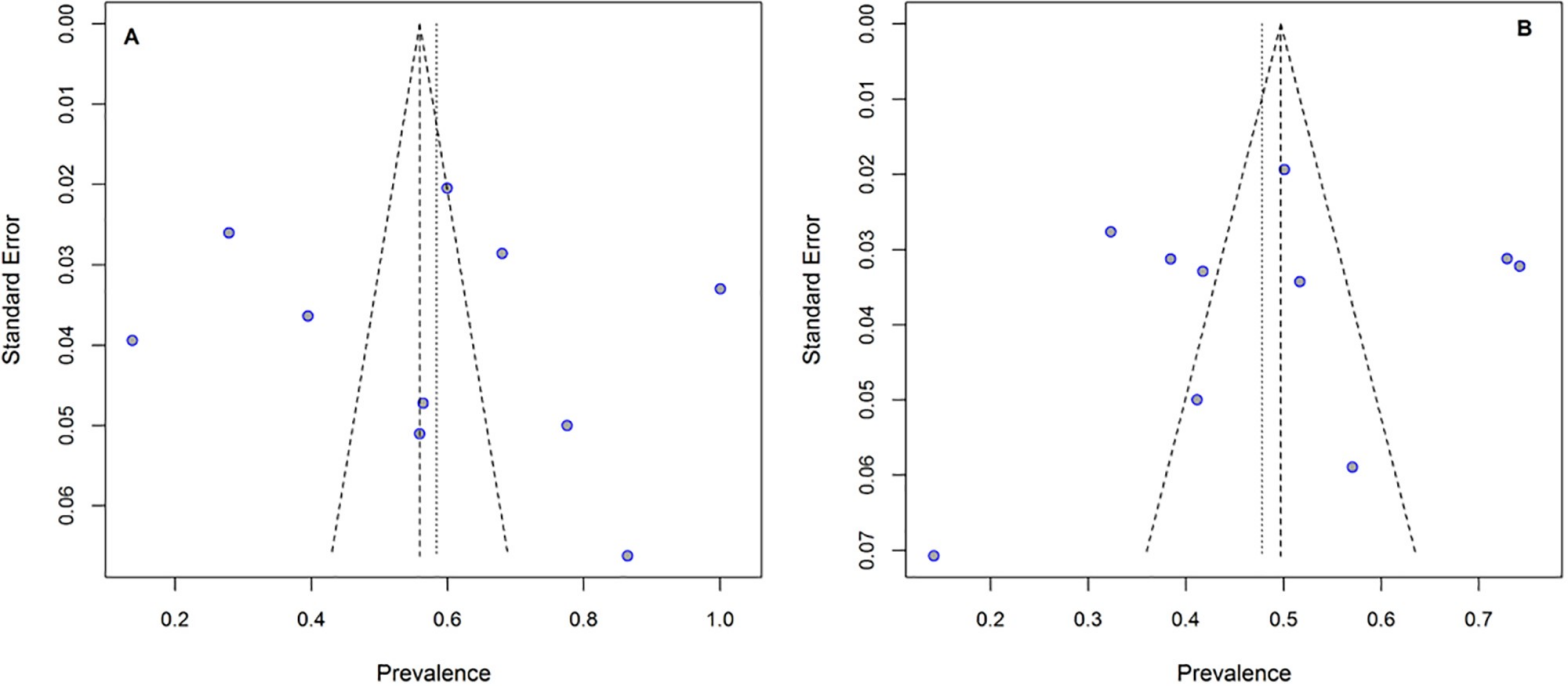
Publication bias using funnel plot. (A) Publication bias in studies with case-control design. (B) Publication bias in studies with cross-sectional design.

Funnel plot was used to identify the potential publication bias. In present study, studies with case-control (Fig 4A, P = 0.58) and cross-sectional (Fig 4B, P = 0.70) design did not have a significant publication bias. According to the Egger's regression test, no significant publication bias was found in studies presenting results for case-control (t = 0.32, P = 0.74) and cross-sectional (t = -0.39, P = 0.70) design.

## 4. Discussion

In general, co-infection of TB and IHIs as well as HIV infection in patients have been a main emerging public health problem in developing countries and in many parts of the world [35–37]. In recent years, although the control of both TB and IHIs has progressed in many parts of the world, there still are millions of new cases of each disease every year [9, 38, 39]. Since there has been no comprehensive investigation on co-infection of TB and IHIs, understanding their epidemiology is crucial for implementing effective control strategies against IHIs in TB patients. Current study is the first meta-analysis carried out to evaluate the prevalence and OR of IHIs in the world population suffering from TB. The results of the present study revealed that IHIs had the overall prevalence of 26% in TB patients both in case-control and cross-sectional studies. According to the findings of the case-control studies, overall pooled OR of IHIs in the cases was not significant compared to the controls (OR, 1; 95% CI, 0–1). However, according to genus/species, the pooled OR of *S*. *stercoralis* (OR, 2.68; 95% CI, 1.59–4.54), had a significantly higher risk in TB patients compared to controls. Among the ten articles included with case-control design, five papers reported a significant difference related with prevalence of IHIs in TB patients [12, 17, 24, 28, 32] in comparison with controls. The number of studies related to this relationship is limited to a few number of countries, which are often African countries. This co-infection is the most common infection in developing and developed countries. Numerous social, economic, geographical, and demographic factors may increase this co-infection [40–42]. Based on the Human Development Index (HDI), African countries are ranked low in comparison with other countries in terms of HDI [43, 44]. Specifically, these countries have the worst condition in terms of poverty index which represents the level of deprivation in three aspects namely life span, education, and income [45, 46]. Our findings showed that the risk of IHI was higher in TB patients compared to controls but this was not statistically significant. However, according to genus/species, the pooled OR of *S*. *stercoralis* had a significantly higher risk in TB patients compared to controls. Since TB agent (Mycobacterium tuberculosis) is an intracellular bacterium, the suppressed immunity in these patients and shift to T helper 2 (Th2) are more susceptible to lead to severe and acute TB. Hence, intestinal helminths are able to strengthen type 2 cells immune response (Th2) via induction of cytokines (e.g. IL-4, IL-5, IL-9, IL-10, and IL-13). Accordingly, change from Th1 toward Th2 during helminths infection can pull down Th1 immune response against MTB[13, 47]. Therefore, helminth infections may play a significant role in the risk of tuberculosis; these infections cannot be easily neglected, especially *S*. *stercoralis*, that can cause dispersed infection in patients with low immunity [48].

Considering that as there is no ‘gold standard’ test (with 100% accuracy) for detection of intestinal helminths; a variety of parasitological methods have been utilized in different areas of the world. However, several diagnostic methods, such as formalin-ether concentration technique, are used as a reliable diagnostic method for helminth eggs, larvae, and protozoan cysts in stool specimens for many laboratories in different parts of the world [49, 50]. On the other hand, direct wet mount is the test commonly used for the diagnosis of intestinal parasitic infections across the world [50]. However, low sensitivity of the direct wet mount technique has been reported in the detection of low intensity infection and this may significantly increase the misdiagnosis of intestinal parasites [49]. Other than these methods, it has been demonstrated that the Kato-Katz and Mini-FLOTAC methods have reasonable accuracy [51, 52]. Due to these problems, a suitable combination of diagnostic methods should be considered in future studies.

In addition, we found a high heterogeneity between studies in this systematic review. The high heterogeneity index is suggestive of potential variation, which could be due to the number of small studies, different diagnostic methods and difference in immune system in humans, which is affected by lifestyle and environmental factors such as dietary habits, environmental pollution, various types of infections, and socioeconomic status [53, 54].

There are several limitations for the current systematic review and meta-analysis: 1) The online registration (PROSPERO) failed because the data were already extracted; 2) there was a low number of research on the prevalence of IHIs in TB patients, and published information on the prevalence of IHIs in TB patients was not available for many parts of the world; 3) in majority of the included articles, related risk factors could not be evaluated such as the mix between adults and children and also male and female; and 4) a huge number of the included case-control studies did not have the precise matching.

It is recommended that a standard questionnaire be designed aiming to conduct a more inclusive judgment on risk factors and clinical symptoms, including sex, age, residence, education, occupation, history of immune suppression, etc.

Finally, future studies need to focus more on those parts of the world which do not have enough data on epidemiological aspects of IHIs in TB patients in order to better understand the overlaps between IHIs and TB patients in endemic countries.

## 5. Conclusion

In this systematic review and meta-analysis, there was a higher risk of hookworm, *A*. *lumbricoides*, and *T*. *trichiura* in TB patients compared to controls based on the type of helminths but this was only significant in the case of *S*. *stercoralis*. There was no association between composite IHIs and TB. Therefore, the results of the current study suggest establishment of a proper health education program in order to take all preventive measures to avoid acquisition of IHIs in TB patients and of TB in IHIs patients. Moreover, it seems that stool examination for IHIs should be included in the routine screening of TB patients.

## Supporting information

S1 File Terms used in search strategies. (PDF)

S1 Fig Forest plot of prevalence of intestinal helminthic infections in case- control and cross-sectional studies. (TIF)

S2 Fig Forest plot of overall prevalence of hookworm. (TIF)

S3 Fig Forest plot of overall prevalence of *Ascaris lumbricoides*. (TIF)

S4 Fig Forest plot of overall prevalence of *Strongyloides stercoralis*. (TIF)

S5 Fig Forest plot of overall prevalence of *Trichuris trichuria*. (TIF)

S6 Fig Forest plot of odds ratios for relationship between prevalence of hookworm, *Ascaris lumbricoides*, *Trichuris trichuria* and TB patients in case-control studies. (TIF)

S7 Fig Forest plot of odds ratios for relationship between prevalence of *Strongyloides stercoralis* and TB patients in case-control studies. (TIF)
