# Supplementary material for: Are intestinal helminths playing a positive role in tuberculosis risk? A systematic review and meta-analysis
Source: PLoS One. 2019 Oct 15;14(10):e0223722. doi: 10.1371/journal.pone.0223722 (PMC6793940; doi:10.1371/journal.pone.0223722)
Supplement: S1 File — (PDF) [file pone.0223722.s001.pdf]

S1 file: Terms used in search strategies.

PubMed and Web of Science

Parasite + Intestinal parasites + *Mycobacterium tuberculosis*

Parasite + Helminth + Tuberculosis

Parasite + Tuberculosis

Parasite + *Mycobacterium tuberculosis*

Parasite + Pulmonary tuberculosis

Parasite + Epidemiology + Tuberculosis

Parasite + Prevalence + *Mycobacterium tuberculosis*

Intestinal parasites + Helminth + *Mycobacterium tuberculosis*

Intestinal parasites + Tuberculosis

Intestinal parasites + *Mycobacterium tuberculosis*

Intestinal parasites + Pulmonary tuberculosis

Intestinal parasites + Epidemiology + *Mycobacterium tuberculosis*

Intestinal parasites + Prevalence + *Mycobacterium tuberculosis*

Helminth + Tuberculosis

Helminth + *Mycobacterium tuberculosis*

Helminth + Pulmonary tuberculosis

Science Direct and Google Scholar

Intestinal parasites+ Infectious disease

Intestinal parasites + Tuberculosis

Intestinal parasites + TB

Intestinal parasites + MTB

Intestinal parasites + *Mycobacterium tuberculosis*

Mycobacterium tuberculosis + Pulmonary tuberculosis

Intestinal infection + *Mycobacterium tuberculosis*

Intestinal infection + Tuberculosis

Tuberculosis + Parasites

Intestinal infection + PTB

Intestinal infection + TB
