# Supplementary figures and images for: Are intestinal helminths playing a positive role in tuberculosis risk? A systematic review and meta-analysis
Source: PLoS One. 2019 Oct 15;14(10):e0223722. doi: 10.1371/journal.pone.0223722 (PMC6793940; doi:10.1371/journal.pone.0223722)

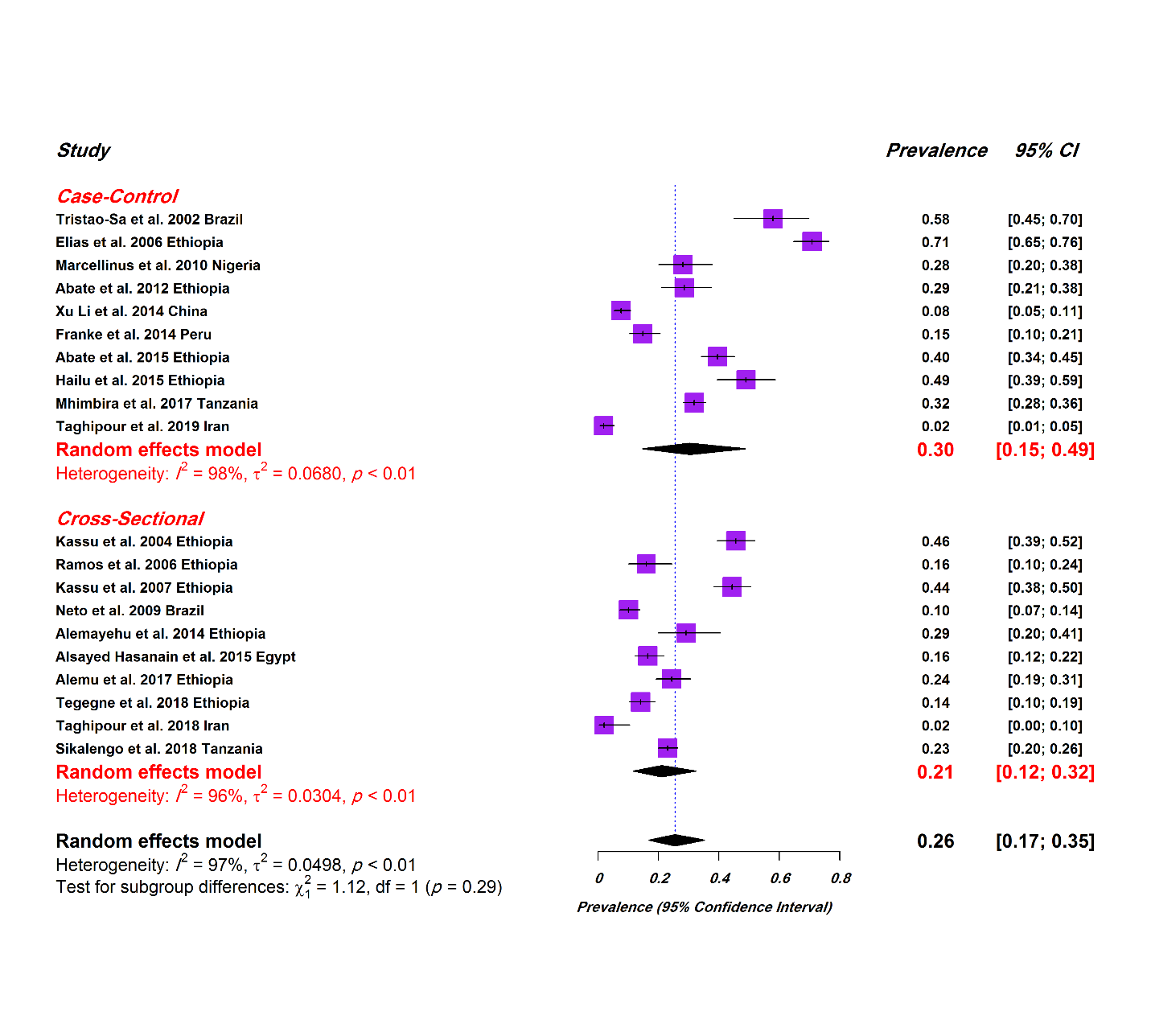

Supplement: S1 Fig — (TIF) [file pone.0223722.s002.tif]

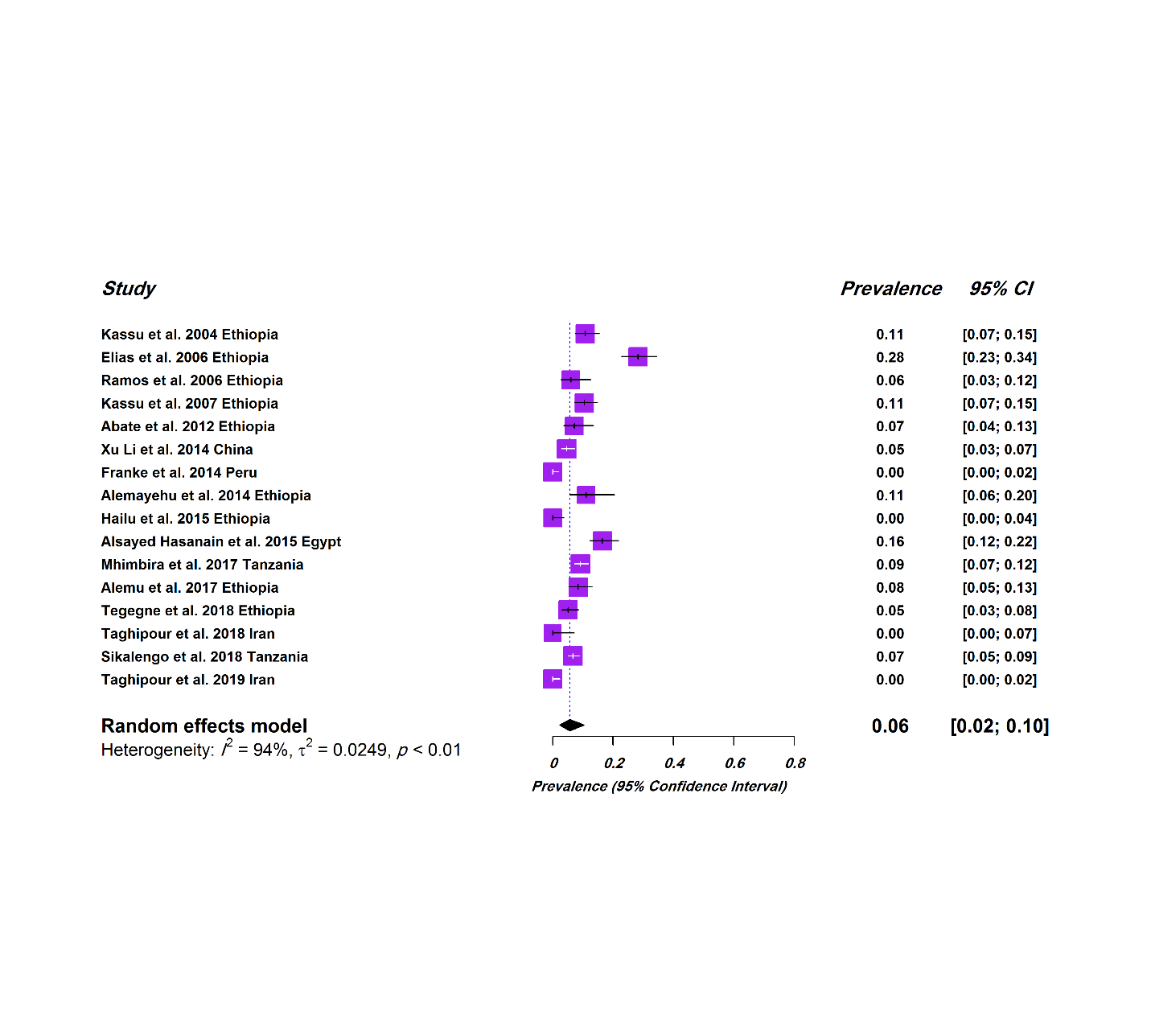

Supplement: S2 Fig — (TIF) [file pone.0223722.s003.tif]

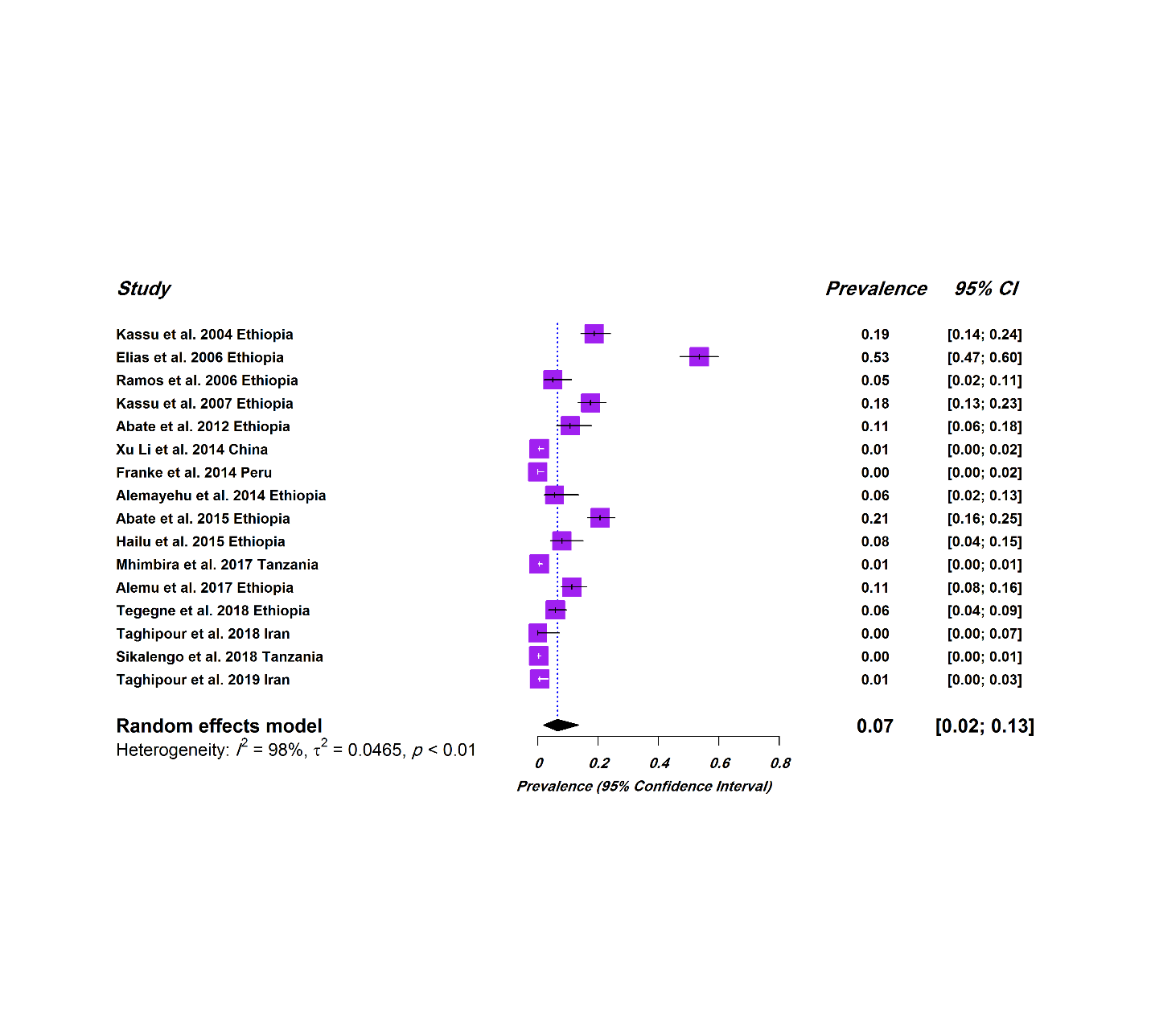

Supplement: S3 Fig — (TIF) [file pone.0223722.s004.tif]

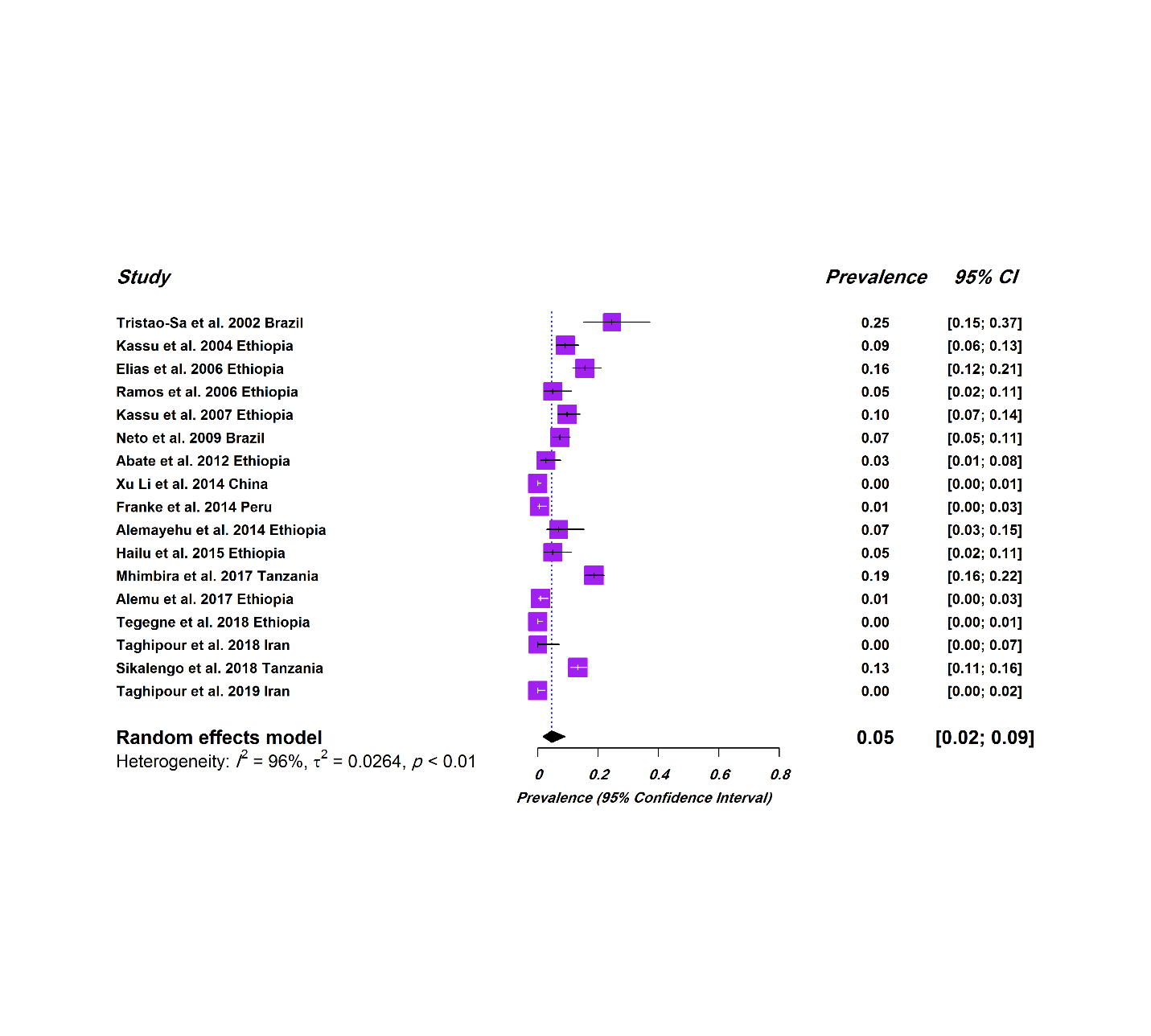

Supplement: S4 Fig — (TIF) [file pone.0223722.s005.tif]

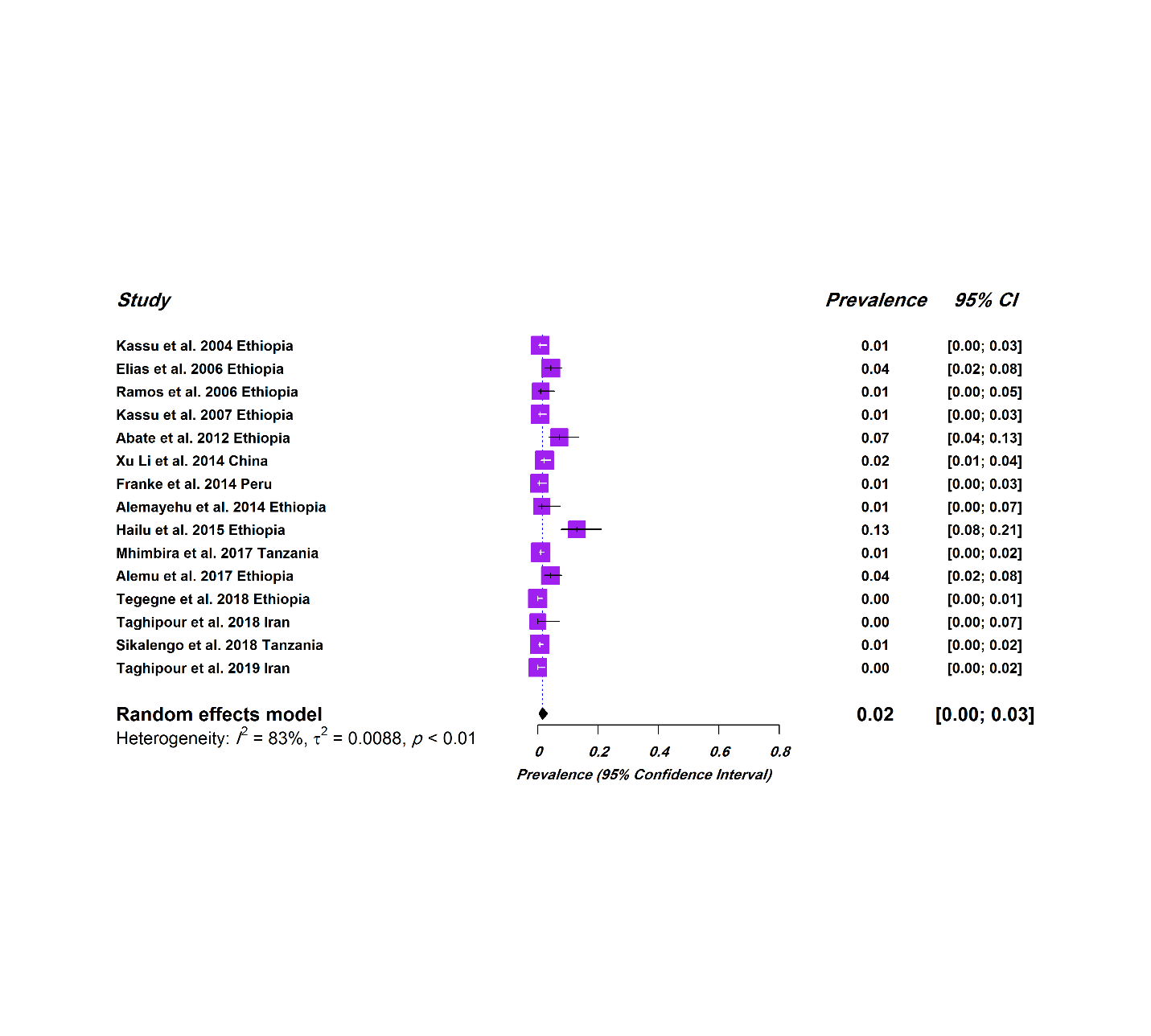

Supplement: S5 Fig — (TIF) [file pone.0223722.s006.tif]

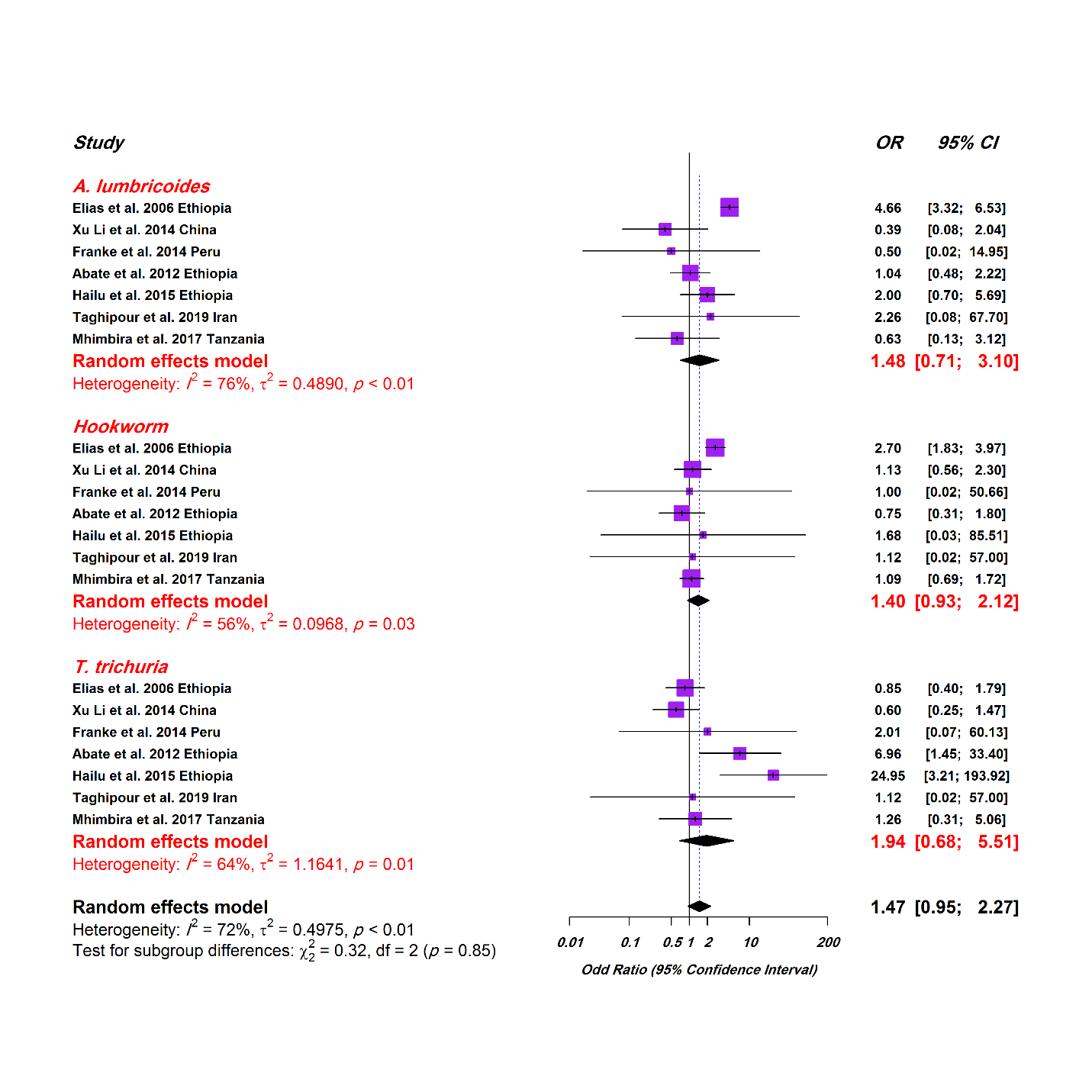

Supplement: S6 Fig — (TIF) [file pone.0223722.s007.tif]

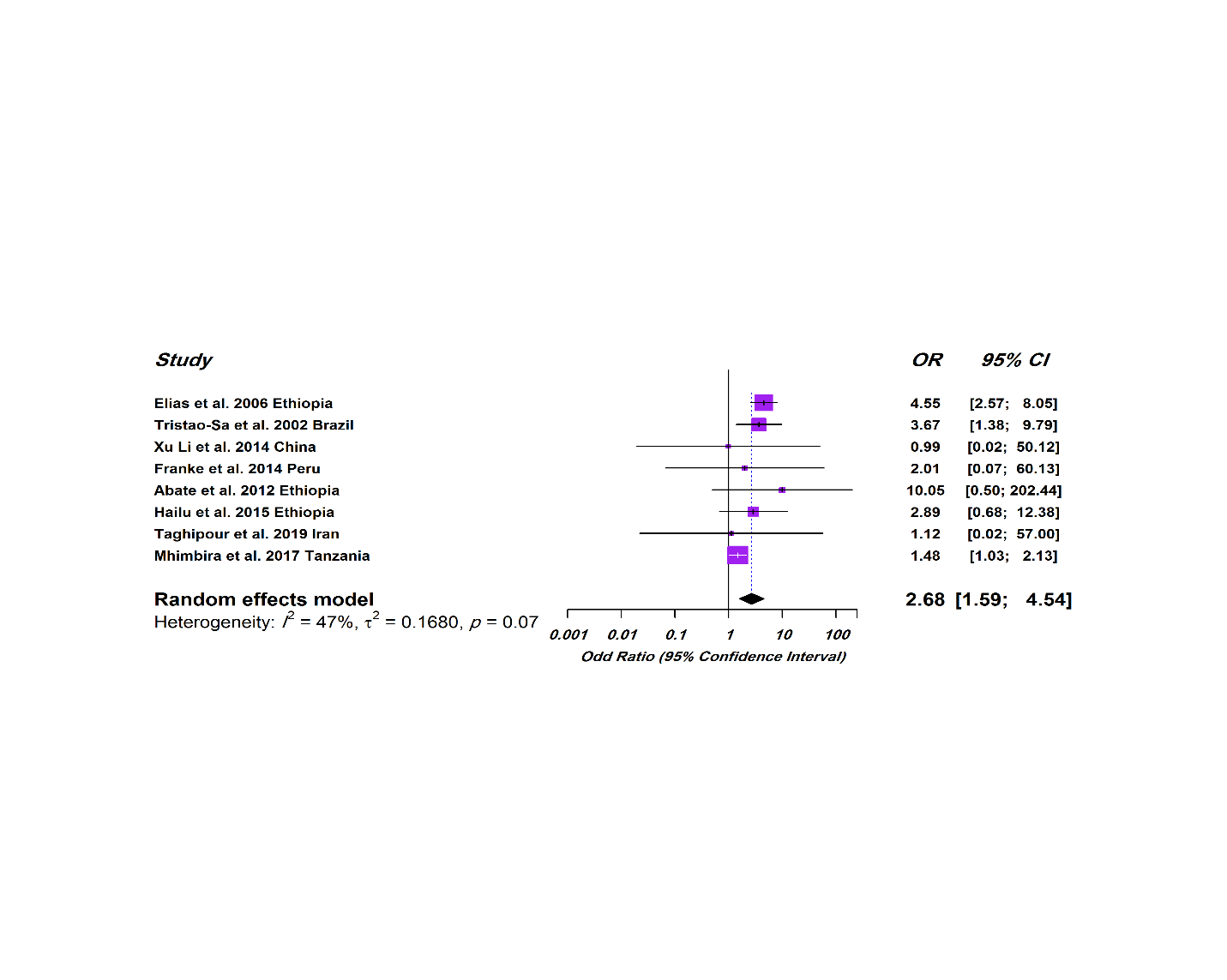

Supplement: S7 Fig — (TIF) [file pone.0223722.s008.tif]
